# Supplementary material for: An open-source framework for large-scale, flexible evaluation of biomedical text mining systems
Source: J Biomed Discov Collab. 2008 Jan 29;3:1. doi: 10.1186/1747-5333-3-1 (PMC2276192; doi:10.1186/1747-5333-3-1)
Supplement: Additional file 1 — Evaluation platform source code. The additional file contains the software evaluation framework discussed in this paper. The most current release can be downloaded from SourceForge.net [1]. See the accompanying README file for instructions on installation and use. Also included in the distribution is a collection of UIMA wrappers for some commonly used BioNLP tools and annotated corpora. [file 1747-5333-3-1-S1.zip › bionlp_uima_repository-1.0.1/docs/api/edu/uchsc/ccp/util/nlp/mention/comparison/ComplexSlotMentionComparator.html]

ComplexSlotMentionComparator (Center for Computational Pharmacology Util/UIMA API)


|  |  |  |
| --- | --- | --- |
| **Overview** \| **Package** \| **Class** \| **Tree** \| **Deprecated** \| **Index** \| **Help** | |  |
| **PREV CLASS**   **NEXT CLASS** | **FRAMES**    **NO FRAMES**     **All Classes** |  |
| SUMMARY: NESTED \| FIELD \| CONSTR \| METHOD | DETAIL: FIELD \| CONSTR \| METHOD |  |


---


## edu.uchsc.ccp.util.nlp.mention.comparison Interface ComplexSlotMentionComparator

**All Known Implementing Classes:**: IdenticalComplexSlotMentionComparator

---

``` public interface ComplexSlotMentionComparator ```

---

| **Method Summary** | |
| --- | --- |
| `int` | `compare(ComplexSlotMention complexSlotMention1, ComplexSlotMention complexSlotMention2, SpanComparator spanComparator, int maximumComparisonDepth, int depth)` |

**Method Detail**

### compare

```
int compare(ComplexSlotMention complexSlotMention1,
            ComplexSlotMention complexSlotMention2,
            SpanComparator spanComparator,
            int maximumComparisonDepth,
            int depth)
```


---


|  |  |  |
| --- | --- | --- |
| **Overview** \| **Package** \| **Class** \| **Tree** \| **Deprecated** \| **Index** \| **Help** | |  |
| **PREV CLASS**   **NEXT CLASS** | **FRAMES**    **NO FRAMES**     **All Classes** |  |
| SUMMARY: NESTED \| FIELD \| CONSTR \| METHOD | DETAIL: FIELD \| CONSTR \| METHOD |  |


---
